# The Association of Renal Agenesis and Ipsilateral Seminal Vesicle Cyst: Zinner Syndrome Case Report

**DOI:** 10.1155/2019/1242149

**Published:** 2019-06-23

**Authors:** Mishal AlArifi, Magdi Al-Gahwary, Mohamed Gomha

**Affiliations:** ^1^College of Medicine, Imam Abdulrahman Bin Faisal University, Dammam, Saudi Arabia; ^2^Department of Urology, King Fahad Specialist Hospital, Dammam, Saudi Arabia

## Abstract

**Introduction:**

Zinner syndrome is a rare congenital malformation characterized by the association of an ipsilateral renal agenesis, ipsilateral seminal vesicle cyst, and ipsilateral ejaculatory duct obstruction. This case is one of the first to be reported in the Kingdom of Saudi Arabia.

**Case Presentation:**

A 20-year-old Saudi male patient presented complaining of chronic left groin pain radiating to the left testis and the medial aspect of the left thigh for the last 6 years. Patient is also complaining of painful ejaculation with no history of lower urinary tract symptoms, hematuria, or trauma. Physical examination was unremarkable. Ultrasound (US) report from the referring hospital mentioned that there is a left pelvic mass. Computed tomography (CT) and magnetic resonance imaging (MRI) showed diffuse distension of left seminal vesicle (9 X 7 cm) cyst with ipsilateral left renal agenesis which corresponds to Zinner syndrome. Left seminal vesicle cyst excision through a low midline incision was done. The patient was asymptomatic during his follow-up in our clinic with disappearance of the pain.

**Conclusion:**

The combination of a good clinical history and radiological assays aided in making the diagnosis. Surgical intervention is the mainstay in the management plan in symptomatic patients.

## 1. Introduction

Zinner syndrome is a rare congenital malformation characterized by the association of an ipsilateral renal agenesis, ipsilateral seminal vesicle cyst, and ipsilateral ejaculatory duct obstruction which was first described by Zinner et al. in 1914 [1]. Zinner syndrome is caused by malformations of the distal portion of the mesonephric duct and is considered the Mayer-Rokitansky-Kuster-Hauser (MRKH) counterpart in males [2]. This case is one of the first to be reported in the Kingdom of Saudi Arabia.

## 2. Case Presentation

**Figure 1:**
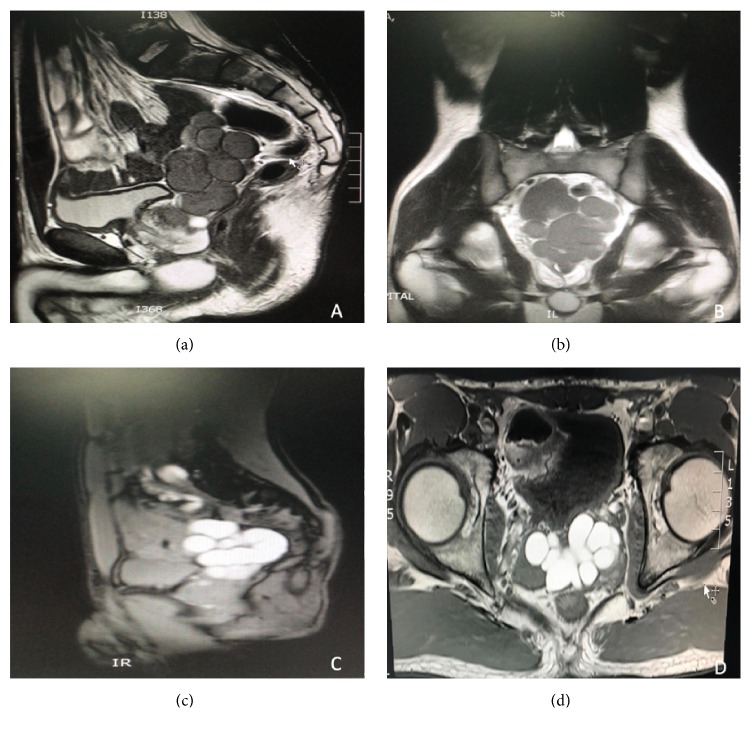
(a) MRI sagittal section T2, (b) MRI coronal section T2, (c) MRI sagittal section T1, and (d) MRI axial section T1. (a)-(b) showed diffuse dilatation of left seminal vesicles with low signal intensity, and (c)-(d) showed diffuse dilatation of left seminal vesicles with high signal intensity.

**Figure 2:**
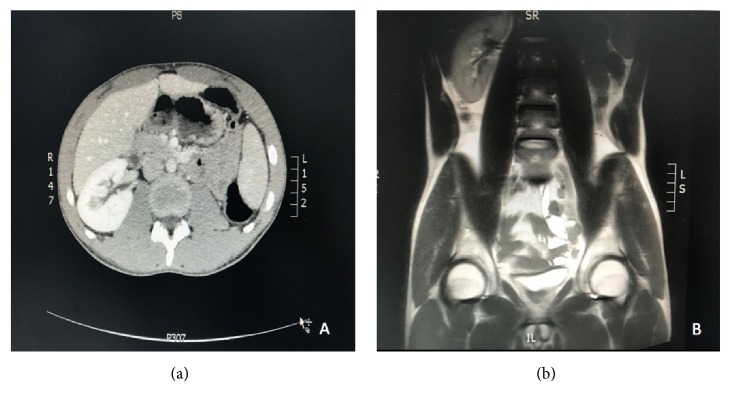
(a) CT axial section showed left renal agenesis. (b) MRI coronal section showed left renal agenesis.

A 20-year-old Saudi male patient not known to have any chronic medical illness was referred to our urology department complaining of chronic left groin pain radiating to the left testis and the medial aspect of the left thigh for the last 6 years. Patient is also complaining of painful ejaculation with no history of lower urinary tract symptoms, hematuria, or trauma. Physical examination was unremarkable. Laboratory tests were ordered including complete blood count and renal function tests which all were within normal ranges. Ultrasound (US) report from the referring hospital mentioned that there is a left pelvic mass. Computed tomography (CT) and magnetic resonance imaging (MRI) showed diffuse distension of left seminal vesicle (9 X 7 cm) cyst (Figure 1) with ipsilateral left renal agenesis (Figure 2) which corresponds to Zinner syndrome. Left seminal vesicle (SV) cyst excision through a low midline incision was done. After general anesthesia and drabbing, a low midline incision was done with a transperitoneal approach, then retracting the intestine exposing and dissecting the right seminal vesicle cysts with release of the adhesion from the pelvic side wall reaching the level of the vas then ligations of both the distal end of SV and vas while preserving the contralateral vas and SV. Haemostasis was done with the insertion of a pelvic drain and closure of the wound in layers. The patient was asymptomatic during his follow-up in our clinic with disappearance of the pain.

## 3. Discussion

Zinner syndrome is one of the rarest congenital anomalies of the urogenital tract and is usually discovered and diagnosed in the 2nd-4th decade of life [3]. The frequency of this condition is reported to be 0.0046% according to Farooqui et al. [4]. Patients with this syndrome are usually asymptomatic but they can present with dysuria, frequency, perineal pain, or epididymitis [3].

The embryological origin of the kidneys and seminal vesicles is similar; the kidney is formed by the metanephric blastema which is induced by ureteral bud that originates from the dorsal aspect of the distal mesonephric duct. The mesonephric duct gives rise to most of the genital tract including epididymis, vas deferens, ejaculatory duct, and seminal vesicle [5]. Any malformation of the ureteral bud or mesonephric duct can cause Zinner syndrome.

Radiological modalities have a significant role in diagnosing and evaluating Zinner syndrome including US, CT, and MRI. US is a quick imaging modality that is used mainly in this condition to detect the absence of the ipsilateral kidney or to show some anechoic structures in the pelvis resembling the obstructed ejaculatory duct [3, 6]. CT findings might include an ipsilateral kidney agenesis in addition to a retrovesicular periprostatic cystic mass [3, 7]. MRI is considered to be the imaging modality of choice in diagnosing this condition due to its high resolution properties in evaluating the seminal vesicles cysts and the ejaculatory ducts [3].

Management of the seminal vesicles cysts depends on the presentation and the presence of symptoms, and asymptomatic patients are usually observed until they start to complain of symptoms. Surgical intervention can be carried out by different modalities and techniques reported in the literature. First option is open surgical excision, and minimally invasive surgeries including the laparoscopic and robotic-assisted approaches are considered the best option due to their better outcomes, less blood loss, and shorter hospitalization [8–11]. Another option is simple cyst drainage or transrectal aspiration which provides a transient relief of symptoms but both are associated with greater chance of recurrence.

## 4. Conclusion

The association of ipsilateral renal agenesis and ipsilateral seminal vesicle cyst is a rare unique congenital presentation that is called Zinner syndrome. The combination of a good clinical history and radiological assays aided in making the diagnosis. Surgical intervention is the mainstay in the management plan in symptomatic patients. Relieving the patient symptoms and improving his quality of life were our main goal which was accomplished.

## References

[1] Zinner A. (1914). Ein fall von intravesikaler Samenblasenzyste. *Wiener Medizinische Wochenschrift*.

[2] Khanduri S., Katyal G., Sharma H. (2017). Unique association of multiple seminal vesicle cysts with contralateral renal agenesis: a rare variant of zinner syndrome. *Cureus*.

[3] Mehra S., Ranjan R., Garga U. C. (2016). Zinner syndrome—a rare developmental anomaly of the mesonephric duct diagnosed on magnetic resonance imaging. *Radiology Case Reports*.

[4] Farooqui A., AlDhahir L., Mahfooz A. B. (2018). Massive seminal vesicle cyst with ipsilateral renal agenesis - Zinner syndrome in a Saudi patient. *Urology Annals*.

[5] Slaoui A., Regragui S., Lasri A. (2016). Zinner's syndrome: report of two cases and review of the literature. *Basic and Clinical Andrology*.

[6] Trigaux J., Van Beers B., Delchambre F. (1991). Male genital tract malformations associated with ipsilateral renal agenesis: sonographic findings. *Journal of Clinical Ultrasound*.

[7] Kenney P. J., Leeson M. D. (1983). Congenital anomalies of the seminal vesicles: spectrum of computed tomographic findings. *Radiology*.

[8] Kord E., Zisman A., Darawsha A. E., Dally N., Noh P. H., Neheman A. (2017). Minimally invasive approach for treatment of seminal vesicle cyst associated with ipsilateral renal agenesis. *Urologia Internationalis*.

[9] Van den Ouden D., Blom J. H. M., Bangma C., De Spiegeleer A. H. V. (1998). Diagnosis and management of seminal vesicle cysts associated with ipsilateral renal agenesis: a pooled analysis of 52 cases. *European Urology*.

[10] Politano V. A., Lankford R. W., Susaeta R. (1975). A 9 transvesical approach to total seminal vesiculectomy: a case report. *The Journal of Urology*.

[11] Okoye B. O., Jones D. J., Lancashire M. J., Brown E. F., Ritchie A. W. S. (1995). Transvesical endoscopic drainage of a seminal vesicle cyst. *British Journal of Urology*.

